# First DNA Sequencing in Beninese Indigenous Cattle Breeds Captures New Milk Protein Variants

**DOI:** 10.3390/genes12111702

**Published:** 2021-10-26

**Authors:** Sèyi Fridaïus Ulrich Vanvanhossou, Isabella Jasmin Giambra, Tong Yin, Kerstin Brügemann, Luc Hippolyte Dossa, Sven König

**Affiliations:** 1 Institute of Animal Breeding and Genetics, Justus-Liebig-University Gießen, 35390 Gießen, Germany; Seyi.F.Vanvanhossou-2@agrar.uni-giessen.de (S.F.U.V.); Isabella.J.Giambra@agrar.uni-giessen.de (I.J.G.); Tong.Yin@agrar.uni-giessen.de (T.Y.); Kerstin.Bruegemann@agrar.uni-giessen.de (K.B.); 2 School of Science and Technics of Animal Production, Faculty of Agricultural Sciences, University of Abomey-Calavi, Abomey-Calavi, 03 BP 2819 Jéricho Cotonou, Benin; hippolyte.dossa@fsa.uac.bj

**Keywords:** casein and whey protein genes, genetic variants, casein haplotype, African taurine and indicine breeds, microRNA, transcription factor binding sites

## Abstract

This study investigated polymorphisms in the milk protein genes *CSN1S1*, *CSN2*, *CSN1S2*, *CSN3*, *LALBA*, and *LGB*, and casein haplotypes in Beninese indigenous cattle. Considering 67 animals, DNA sequencing of the genes’ exons, flanking regions and parts of the 5′-upstream regions identified 1058 genetic variants including 731 previously unknown. In addition, four novel milk protein variants were detected, including *CSN3^K^* (p.Ala66Val), *LALBA^F^* (p.Arg58Trp), *LGB^B1^* (p.Ala134Val) and *LGB^K^* (p.Thr92Asnfs*13). *CSN3^K^* is caused by a novel SNP (BTA6:85656526C>T, exon 4) whereas *LALBA^F^* and *LGB^B1^* are due to rs714688595C>T (exon 1) and rs109625649C>T (exon 4), respectively. Regarding *LGB^K^*, a frameshift insertion of one adenine residue at BTA11:103257980 (exon 3) induces a premature translation termination resulting in a 46% reduction of the reference protein sequence. The casein polymorphisms formed five main *CSN1S1-CSN2-CSN1S2-CSN3* haplotypes including B-A1-A-B, B-A1-A-A and C-A2-A-B which are predominant in the investigated cattle breeds. Moreover, in silico analyses of polymorphisms within the 5′- and 3′- untranslated regions of all six milk proteins revealed effects on microRNA and transcription factor binding sites. This study suggests a large genetic variation of milk protein genes in Beninese cattle, which should be investigated in further studies for their effects on milk production, including quality and yield traits.

## 1. Introduction

The dairy sector in Benin, as in many African countries, is characterized by low productivity and strong dependence on imports to meet the growing demand for dairy products [1,2]. To address this situation, governments and farmers are opting for exogenous breeds, perceived as more productive. For example, the Girolando and Azawak breeds were introduced in Benin for crossbreeding in order to improve herd productivity [2,3]. Also, crossbreeding between local taurine and zebu breeds is increasingly practiced by small-scale farmers [4]. However, neither selection strategies nor proper documentation on the characteristics of the local breeds exist as a basis for optimized crossbreeding practices [5,6]. Imported exotic breeds are challenged due to their inferior adaptation to local harsh environments, implying increased disease frequencies and ongoing declines in productivity. A focus on exotic breeds also implies a growing risk to lose specific values and genetic diversity characterizing the local breeds [1,2]. Indeed, the taurine cattle (Somba, Lagune) are typically acknowledged for the appreciable organoleptic and cheesemaking qualities of their milk implying high economic values [7,8]. In contrast, the zebu cattle are known for higher milk yield, but of poorer milk quality [7]. As argued by Said Ahmed et al. [9], conservation and breeding strategies to improve milk yield should consider the maintenance of genetic diversity and conservation of specific traits of interest, including nutritional, immunological and technological quality of milk.

Six milk protein genes influence milk production traits. They include four casein genes (*CSN1S1*, *CSN2*, *CSN1S2*, *CSN3*) on *Bos taurus* autosome (BTA) 6 and two whey protein genes (*LALBA*/*LAA* and *LGB*/*PAEP*) on BTA5 and BTA11, respectively [10,11]. The genes encode the proteins α_s1_-casein (CN), β-CN, α_s2_-CN, κ-CN, α-Lactalbumin (α-LA), and β-Lactoglobulin (β-LG), respectively. The four caseins form a 250-kb cluster resulting in a haplotype on BTA6 [12]. Protein electrophoresis, mRNA- and DNA-sequence analyses identified enormous variability within the six main milk proteins [13,14]. The protein polymorphisms described in bovine milk include to date 10 protein variants for *CSN1S1* (A, B, C, D, E, F, G, H, I, J), 15 protein variants for *CSN2* (A1, A2, A3, B, C, D, E, F, G, H1, H2, I, J, K, L), five protein variants for *CSN1S2* (A, B, C, D, E), 14 protein variants for *CSN3* (A, A1, B, B2, C, D, E, F1, F2, G1, G2, H, I, J), five protein variants for *LALBA* (A, B, C, D, E), and 11 protein variants for *LGB* (A, B, C, D, E, F, G, H, I, J, W) [14,15,16]. Some protein variants, including the *CSN1S1^H^* and *CSN3^J^*, are exclusively described in the West-African taurine breed Baoulé and the crossbreed Kuri, respectively [17]. Regarding the Beninese cattle breeds, only two studies addressed milk protein gene variability [18,19]. Both studies considered samples collected in 1997 and 1998, and described divergent distribution of milk protein variants in the breeds, especially between the taurine (Lagune and Somba) and the crossbreed (Borgou) [18,19]. An unknown variant α_s1_-CN^X^ identified by Moazami-Goudarzi et al. [18] in Borgou animals is not clarified on DNA or mRNA level to date. Several investigations on cattle breeds in other countries associated milk protein variants with milk yield, composition and coagulation traits [20,21,22]. For instance, the β-CN^A2^, predominant in zebu cattle presented positive effects on calf growth [23]. This variant is also of high interest for human health because of its ability to promote better digestion efficiency and antibacterial activity [14,17,24]. In contrast, the β-CN^A1^ and κ-CN^B^ variants, observed in high frequency in the Beninese taurine breeds, have been linked to better cheesemaking properties (curd consistency, milk or rennet coagulation, micelle size). These descriptions match the appreciable features of Beninese taurine milk as described by the farmers [25,26]. Therefore, in Beninese cattle, we hypothesize a high correlation between milk production with milk protein genetic variability.

In addition to single milk protein variants, casein clusters significantly affected milk characteristics in ruminants [27]. Researchers reported significant relationships between different haplotypes and milk protein yield, coagulation, cheesemaking properties and offspring performances [28,29]. Braunschweig et al. [30] compared the parental haplotypes B-B-A-A and B-B-A-B for *CSN1S1-CSN2-CSN1S2-CSN3* in Swiss Brown Cattle and associated B-B-A-A with lower casein content and higher whey protein content in daughter milk. Similarly, the maternal composite casein genotype *CSN1S1*^BB^*-CSN2*^A2A2^*-CSN1S2*^AA^*-CSN3*^AB^ have been recently associated with pre-weaning offspring growth in German cattle [23].

Furthermore, polymorphisms occurring in the promoter regions of the milk protein genes are known to affect the binding sites of transcription factors (TF) or microRNA (miRNA) [31]. Transcription factors are proteins that bind to a specific DNA sequence (transcription factor binding sites, TFBS) in the cis-regulatory/intergenic regions to modulate the transcription or expression of a gene [32,33]. Similarly, miRNA, small non-coding RNA, interact with the 5′- and 3′-untranslated regions (UTR) and with gene promoter regions to suppress or activate expression, or control the translation and transcription rate of a gene [34,35,36]. Various single nucleotide polymorphisms (SNP) are located in TFBS or miRNA target sites flanking the milk protein genes, and are implicated in the alteration or reduction of milk protein gene functions (e.g., resulting in lower protein content) [9,37,38,39]. For example, the g.-475C>G SNP positioned within the activator protein-2 (AP-2) TFBS was associated with differential expression of *LGB^A^* and *LGB^B^* [40,41]. Likewise, SNP-haplotypes in the promoter regions of the *CSN1S1* are linked with different promoter alleles affecting milk production traits [42,43]. Hence, investigating the exons and flanking regions of milk protein genes in Beninese indigenous cattle breeds is beneficial to increase knowledge on existing genetic variation and on ongoing evolutionary patterns, to unravel their potential impacts in milk production, and to establish appropriate management strategies [9,44].

Screening milk protein polymorphisms and genotype composites and casein haplotypes is particularly important in Benin given the increasing crossbreeding and environmental pressures [45,46]. Caroli et al. [14] recommended frequent monitoring of milk protein genetic variability in cattle populations to detect and prevent the expansion of undesirable genetic mutations with unfavorable effects on milk traits. Gallinat et al. [15] recently identified new milk protein variants, reflecting the evolutionary pattern in different cattle breeds. With regard to the variety of tools to assess genetic polymorphisms in milk protein genes and haplotypes (at protein or DNA level), genomic methods provide a wider overview of chromosome variations [14,23]. The latter approach is increasingly being used to characterize known and novel polymorphisms in cattle milk protein genes [9,14,47]. Consequently, in the present study, we performed the first DNA sequencing of Beninese cattle, focusing on exons and flanking intron regions and parts of the 5′-upstream regions of milk protein genes, and we explored their functional consequences in silico. Milk protein polymorphisms and casein haplotypes were subsequently characterized in the Beninese indigenous cattle breeds.

## 2. Materials and Methods

### 2.1. Animal Sampling, DNA Extraction and Sequencing

Sixty-seven animals consisting of 20 Lagune, 20 Somba, 27 crossbreeds (including Borgou and Pabli animals) were selected within a large dataset of Beninese indigenous cattle sampled between 2016 and 2017 [45]. The Lagune and the Somba are two representatives of West African taurine, while the Borgou and Pabli are the products of crossbreeding, i.e., Somba × White Fulani (Zebu) and Somba × Borgou, respectively [6]. The relationships between the animals were minimized by considering only one sample per herd and per village. In addition, purebred Lagune and Somba animals with respective Lagune and Somba ancestry proportions ≥ 87% were selected, considering the values estimated with Admixture by Scheper et al. [45]. 

The DNA of the selected animals was extracted from hair samples using the NucleoSpin Tissue Kit (MACHEREY-NAGEL GmbH & Co. KG, Düren, Germany), following manufacturer’s instructions. A minimum of 20 μL of DNA with a concentration of ~30 ng/μL were submitted to LGC Genomics GmbH (Berlin, Germany) for the sequencing of all exons and flanking intron regions as well as of parts of the 5′-regions of the casein and whey protein genes. The primer sequences described in Said Ahmed et al. [9] were used to amplify all exon sequences of the six milk protein genes under investigation. Moreover, additional primer pairs were established to amplify and subsequently sequence slightly larger 5′-upstream regions of the *CSN1S1*, *CSN3*, *LALBA* and *LGB* genes, respectively (see Table S1). Sequencing was made via Genotyping-in-Thousands by sequencing (GT-seq) using the Illumina MiSeq V3 platform with 2 × 300 bp paired-end read (LGC Genomics GmbH, Berlin, Germany). Using this method, next-generation sequencing of multiplexed polymerase chain reaction (PCR) products generated genotypes for a panel of DNA polymorphisms, in our case in the six main milk proteins in cattle.

### 2.2. Processing of Sequence Data

The raw sequence data consisting of 5 million reads were de-multiplexed with the Illumina bcl2fastq v2.20 [48]. Quality control using the FastQC v0.11.9 software filtered out low quality reads (containing ambiguous base, with final length inferior to 65 bases or with an average Phred quality score inferior to 30 over a window of ten bases). Subsequently, the BWA-MEM algorithm [49] was used to align the quality trimmed sequence reads against the respective reference sequences of cattle milk protein genes from the Ensembl genome database (http://www.ensembl.org/Bos_taurus/, accessed on 13 October 2021, see Table 1). Finally, variants and genotypes covered by a minimum coverage of 8 reads were called using the Bayesian haplotype-based method implemented in Freebayes v1.2.0, setting ploidy equal to 2. The outputs were checked and multiple nucleotide polymorphisms were manually processed to retrieve SNP, insertions and deletions (InDel).

### 2.3. Functional Annotation of DNA Sequence Variants, Frequencies of SNP Alleles and Casein Haplotypes

The positions of the identified sequence variants were inferred considering the gene sequence positions in the ARS-UCD1.2 genome assembly. Functional annotation, performed with the Ensembl Variant Effect Predictor online tool [50], retrieved known variants (referenced in the Ensembl database) and their potential consequence. The previously undescribed DNA variants were submitted to the European Variation Archive (EVA) which assigned individual variant accessions (ss#) number to them [51]. To avoid redundancies and discrepancies in the functional annotation of the polymorphisms, only the most conserved transcript was investigated for each milk protein gene (i.e., ENSBTAT00000010119.3 for *CSN1S1*, ENSBTAT00000003409.6 for *CSN2*, ENSBTAT00000006590.6 for *CSN1S2*, ENSBTAT00000028685.5 for *CSN3*, ENSBTAT00000007701.2 for *LALBA* and ENSBTAT00000019538.6 for *LGB*). Variants detected in the coding regions were considered to annotate milk protein variants following the existing nomenclature [9,14,15,52] and referring to the amino acid (AA) position in the mature proteins.

SNP allele frequencies in the respective breeds were calculated with the vcftools v0.1.16 software [53]. In addition, haplotypes were constructed across the casein cluster *CSN1S1-CSN2-CSN1S2-CSN3*, and their frequencies were estimated using the haplo.group function in the haplo.stats (version 1.8.7) R package [54]. In this regard, we estimated the maximum likelihood of haplotype probabilities in the different breeds as well as for the whole population. Only the casein variants with a minimum allele frequency of 5% in the overall population were considered for haplotype constructions.

Potential effects of the splice site variants were investigated in silico using the NNSPLICE 0.9 tool [55]. In addition, the polymorphisms located in the 5′- and 3′-UTR of the milk protein genes were examined for the prediction of TFBS and miRNA targets. TFBS were identified applying the sequence analysis workflow with the Gene Transcription Regulation Database (GTRD) [56,57] in the geneXplain platform 6.4 web server (https://platform.genexplain.com/bioumlweb/, accessed on 13 October 2021). For the investigation of miRNA target sites, the seed regions of bovine miRNA were downloaded on the TargetScan website (http://www.targetscan.org, accessed on 13 October 2021, see Table S2) and used applying the “targetscan_60.pl” algorithm [58,59].

## 3. Results

### 3.1. Distribution of DNA Sequence Variants

**Figure 1:**
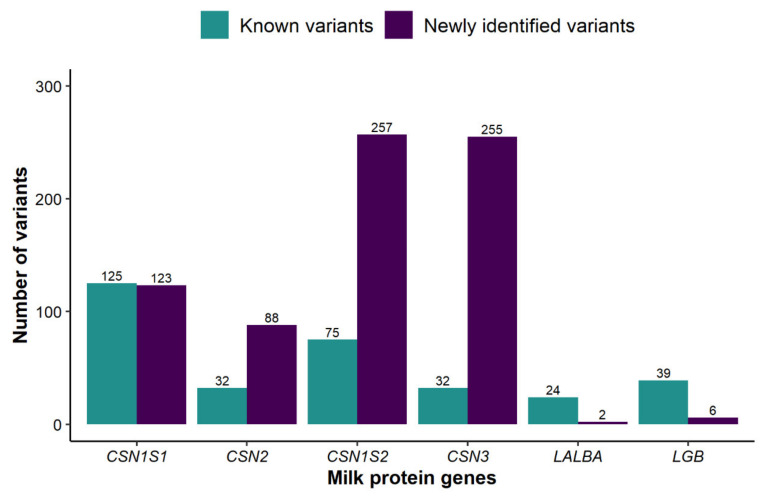
Distribution of known and novel DNA variants within the exon sequences, flanking intron sequences and parts of the 5′-upstream regions of milk protein genes (*CSN1S1*, *CSN2*, *CSN1S2*, *CSN3*, *LALBA*, *LGB*) in Beninese indigenous cattle breeds.

Sequencing of all exon sequences, flanking intron regions and parts of the 5′-upstream regions of the six milk protein genes, and subsequent comparisons with the Ensembl bovine reference sequence (Table 1), displayed a total of 987 SNP and 71 InDel. With regard to the total number of variants identified in the respective gene, the *CSN1S1, CSN1S2* and *LALBA* presented the highest proportions of InDel (Table 2). Only 30.91% (327 variants) of the detected polymorphisms were referenced in the Ensembl variant table, whereas 731 variants have not been reported before (Table S3). The latter variants include 21 SNP occurring at the same position of a known SNP (in the Ensembl variant table), but with new alleles (Table S3). In comparison to the other genes, *CSN3* displayed the highest proportion (88.85%) of newly identified variants (Figure 1). In contrast, the majority of the identified variants in *LALBA* and *LGB* are already referenced in the Ensembl variant table. 

**Figure 2:**
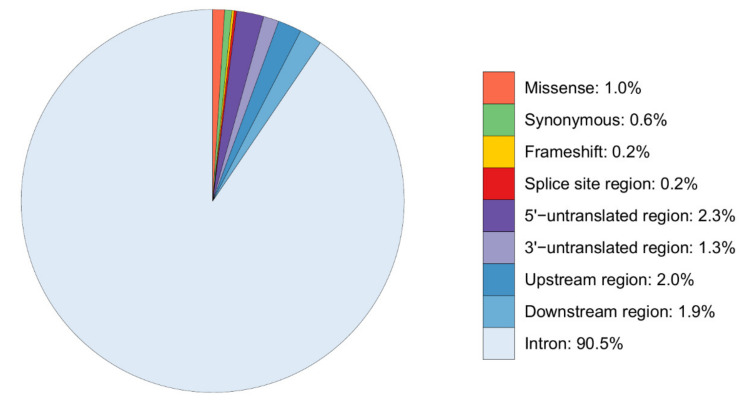
Overall percentage of the DNA variant types detected within the exon sequences (missense, synonymous, frameshift), flanking intron sequences and parts of the 5′-upstream regions of the six milk protein genes (*CSN1S1*, *CSN2*, *CSN1S2*, *CSN3*, *LALBA*, *LGB*) in Beninese indigenous cattle breeds. # The respective distributions of variant types for each gene (see above Figure 1) are presented in Table S4.

The variants were mainly detected in the intron regions (90.5%), 5′-UTR (2.3%), 5′-upstream (2%) and 3′-downstream (1.9%) regions (Figure 2). Only 2% of the detected variants were positioned in coding regions (missense, synonymous and frameshift variants). The *CSN3* and *LALBA* genes displayed the majority of the missense variants (three SNP for each of the genes). A frameshift deletion and insertion were identified in *LALBA* and *LGB,* respectively. In addition, a total of six synonymous SNP were detected in *CSN2*, *CSN3*, *LALBA* and *LGB* (Table S4). 

### 3.2. Causal Genetic Polymorphisms of Milk Protein Variants

A total of 11 missense SNP and one frameshift insertion have been identified in the milk protein genes in the investigated population. Table 3 presents the identified sequence differences, their locations within the respective gene, their effects on the protein sequence, the protein variant designations and frequencies within the different breeds and across all Beninese cattle. More detailed descriptions of the sequence differences found in the respective genes are given in Section 3.2.1 and in the following.

#### 3.2.1. CSN1S1

The sequencing of the 19 exons of the *CSN1S1* gene identified the already known casein variants *CSN1S1^B^* and *CSN1S1^C^*. The latter protein variant is due to the SNP rs43703010C>T located in exon 17, inducing the substitution of the glutamic AA by glycine (p.Glu192Gly) in the mature protein. In our animal material, 81% of all animals, especially the taurine, had the reference allele rs43703010A, leading to *CSN1S1^B^*. In contrast, the alternative allele rs43703010G, causing *CSN1S1^C^*, was mainly present in the crossbreed animals with an intra-breed frequency of 38% (Table 3).

#### 3.2.2. CSN2

We detected the casein variants *CSN2^A1^, CSN2^A2^* and *CSN2^L^*. *CSN2^A2^* is caused by rs43703011A>C in exon 7 (SNP), inducing the replacement of histidine by proline at position 67 of the mature protein (p.His67Pro, Table 3). The allele rs43703011C was dominant in the crossbreeds (intra-breed frequency of 61%). In contrast, the reference allele rs43703011A, leading to the casein variant *CSN2^A1^*, was prevalent in the taurine, especially for Lagune (intra-breed frequency equal to 80%). The casein variant *CSN2^L^* is characterized by a replacement of valine by alanine (p.Val197Ala), due to the SNP rs715383373T>C in exon 7. The allele rs715383373C was identified in one crossbreed animal. Additionally, we identified the SNP rs468218273C>T affecting the AA 42 (p.Ala42) of the immature protein, located within the signal peptide.

#### 3.2.3. CSN1S2

Only the SNP rs441966828C>T (exon 3) differing the protein variants *CSN1S2*^A^ and *CSN1S2^B^* was found in the investigated Beninese cattle population. Indeed, p.Ser8Phe is the resulting AA exchange differentiating *CSN1S2^B^* from *CSN1S2^A^*. The reference SNP allele rs441966828C encoding for *CSN1S2^A^* was observed in the majority (99%) of the animals, while the allele rs441966828T producing the protein variant *CSN1S2^B^* was detected in one crossbreed animal (Table 3).

#### 3.2.4. CSN3

The analysis of the five exons of *CSN3* detected two known (*CSN3^A^*, *CSN3^B^*) and one new casein variant (*CSN3^K^*). The casein variants *CSN3^A^* and *CSN3^B^* are caused by the SNP rs43703015T>C and rs43703016C>A in exon 4, inducing the AA replacements p.Ile136Thr and p.Ala148Asp, respectively. Most of the animals carried the reference SNP alleles rs43703015T and rs43703016C, coding for *CSN3^B^* (Table 3). In contrast, the respective alleles rs43703015C and rs43703016A, coding for *CSN3^A^*, were mainly observed in heterozygous genotypes in the crossbreed animals (50%). Additionally, we detected the synonymous SNP rs110014544G>A (p.Ala168) also differentiating the casein variants *CSN3^A^* and *CSN3^B^*. The novel kappa-casein variant (*CSN3^K^*) detected in this study is due to a previously undescribed SNP (BTA6:85656526C>T) in exon 4 leading to an AA exchange from alanine to valine (p.Ala66Val). The new variant *CSN3^K^* was observed in one crossbreed animal.

#### 3.2.5. LALBA

Across the four exons of *LALBA*, three missense SNP causing four milk protein variants including three known (*LALBA^A^*, *LALBA^B^*, *LALBA^E^*) and one previously undescribed (*LALBA^F^*), were identified. The SNP rs722550244G>C in exon 1 is responsible for the change of arginine to glutamine (p.Arg10Gln), differentiating *LALBA^B^* from *LALBA^A^*. The reference SNP allele rs722550244G causing *LALBA^B^* was present in most of the animals (95%), whereas the alternative SNP allele rs722550244A leading to *LALBA^A^* was only observed in crossbreed animals (Table 3). The protein variant *LALBA^E^* is caused by an A to G nucleotide transition in exon 2 (SNP rs465119286A>G). The SNP allele rs465119286G found in 7% of the animals (including Somba and crossbreed) causes the replacement of isoleucine to valine (p.Ile41Val). In addition to the SNP rs722550244G>C that distinguishes *LALBA^B^* from *LALBA^A^*, the SNP rs7144688595C>T also affects the AA 10 of the mature protein α-LA. Here, the SNP leads to the AA exchange p.Arg10Trp. Therefore, we have introduced a new naming *LALBA^F^* for this protein variant. The SNP allele rs7144688595T was observed in 6% of the animals. Moreover, a synonymous SNP rs477959124C>T (p.Asp64) was identified in exon 2.

#### 3.2.6. LGB

Concerning the *LGB* gene, we identified three milk protein variants (*LGB^B^, LGB^B1^, LGB^K^*) but only *LGB^B^*, being the reference allele (Table 1) was previously described. Indeed, only one causal genetic polymorphism, i.e., the missense SNP rs109625649C>T differing the protein variants *LGB^B^* and *LGB^A^*, was detected in exon 4 (Table 3). The SNP allele rs109625649T observed in 20% of the animals induces an exchange of alanine to valine (p.Ala118Val). With the absence of the second missense SNP characterizing *LGB^A^*, we considered the observed sequence as an intermediate and suggested a new variant name *LGB*^*B*1^. The second novel protein variant *LGB^K^* is caused by a frameshift insertion of an adenine nucleotide at position BTA11:103257980 in exon 3. This new polymorphism was detected in one animal and induced the emergence of a premature stop codon, reducing the coding sequence of the *LGB* gene from a total of 178 to 104 AA with a complete exchange of the protein sequence from AA 92 to 104 (p.Thr92Asnfs*13, see Table S5). Additionally, three synonymous SNP were mapped in the *LGB*, including rs109116595T>C (p.Ile2) in exon 1, rs110641366T>C (p.Asn88) and rs715512468C>T (p.Thr97) in exon 4.

### 3.3. Casein Haplotypes 

A total of 24 casein haplotypes (*CSN1S1-CSN2-CSN1S2-CSN3*) were built from the identified casein variants (with individual allele frequency ≥ 5%). However, only five haplotypes display a frequency of 5% in at least one breed (Table 4). The haplotype B-A1-A-B was the most frequent, especially in Somba (47%) and Lagune (50%). In contrast, the haplotypes B-A1-A-A and C-A2-A-B were predominant in the crossbreeds. Moreover, the B-A2-A-B haplotype was observed in all breeds with comparable frequency.

### 3.4. Functional Effects of Variants in the Splice Sites, 5′- and 3′-Untranslated Regions of Milk Protein Genes

In silico evaluations of the polymorphisms in the non-coding exonic regions of the six milk protein genes displayed various potential effects. The SNP rs452830840C>T and rs208412793G>T are positioned in the splice site regions of *CSN3* and *LGB*, respectively. However, they presented no impact on the binding score of the splice enzymes using NNSPLICE 0.9. Furthermore, no further identified SNP or InDel affected the 5′-donor or 3′-acceptor splice sites of the exons of the six genes. 

The *CSN1S1* and *LALBA* genes presented several SNP in the 5′- and 3′-UTR and many of them influenced TFBS or miRNA target sites (Table 5). In contrast, we mapped only one SNP (rs440770944C>A) in the 3′-UTR of *CSN2*. This SNP altered the binding site of the bta-miR-2464-3p miRNA, but favored the p63 TFBS. Overall, 11 and 15 SNP located in the 5′-UTR of the six milk protein genes affected TFBS or miRNA target sites, respectively. Six SNP were responsible for the loss of TFBS, whereas seven SNP promoted the gain of TFBS. They included one 5′-UTR SNP (rs109900747T>C) causing the loss and the gain of TFBS simultaneously. Five SNP abrogated miRNA target sites in the 5′-UTR, while five other SNP introduced new miRNA target sites. In addition, simultaneous loss and gain of miRNA target sites were observed in five SNP located in the 5′-UTR. Within the 3′-UTR of the milk protein genes, five and 10 SNP altered TFBS and miRNA target sites, respectively. In contrast, two and five SNP introduced new TFBS and miRNA target sites, respectively.

## 4. Discussion

In this study, we sequenced the exons, flanking intron regions, and parts of the 5′-flanking regions of the six milk protein genes from 67 animals, and identified a total of 1058 DNA polymorphisms. This value is higher than those reported in previous studies targeting sequence data of cattle milk protein genes [9,60]. For instance, Meier et al. [60] considered the 1000 Bull Genomes with 1821 animals from 14 different breeds and reported only 892 SNPs within the four casein genes (exons and flanking intron regions) and their 1000 bp upstream regions. In contrast, the four casein genes display 987 variants in our study. This difference implies the high level of polymorphisms in the milk protein gene segments of the Beninese breeds compared to other cattle breeds. In addition, the large number of novel variants is typical in the context of the pronounced genetic diversity of the Beninese cattle, and concurs with the need for deeper investigation of African livestock genomes [45,61]. 

Similar to Meier et al. [60], we observed the majority of the polymorphisms in the intron regions. However, several variants are also located in the coding regions as well as in the 5′- and 3′-UTR. We only focus on the polymorphisms in 5′- and 3′-UTR for the screening of TFBS or target sites for miRNA because of the large number of detected SNP. The identified effects of the 5′- and 3′-UTR SNP on TFBS and miRNA sites (see Table 5) are consistent with previous findings and suggest further investigations on the SNP located in the 5′-upstream and 3′-downstream region of the milk protein genes [9,38]. The analyses of the SNP impacts on the splice site recognition of the milk protein genes using NNSPLICE revealed no effect. However, supplementary tools (e.g., ESEfinder) can be applied to investigate exonic splicing enhancers in the milk protein gene [62]. Moreover, further research is needed to clarify the real effects of these polymorphisms in the TFBS, miRNA binding sites and splice site regions, for example using PCR-based expression studies (like real-time quantitative PCR or droplet digital PCR), gene expression array or RNA sequencing (RNA-Seq) methods [63,64,65,66].

Within the coding regions, a total of 12 DNA polymorphisms caused milk protein variants. They include two previously unknown DNA variants, i.e., one SNP in *CSN3* and one frameshift insertion in *LGB*. The total number of missense variants is appreciable, but less than those (a total of 20 missense SNP in casein genes) reported in the 1000 Bull Genomes consisting of a large variety of cattle breeds [60]. Nevertheless, the higher number of missense variants identified in *CSN2*, *CSN3* and *LALBA* in the present study are correlated with the high polymorphisms of these milk protein genes compared to the others [14,60]. Several other segregating variants of the bovine milk protein genes were also not identified [14,15]. Overall, 17 milk protein variants were characterized and included two for *CSN1S1* (B, C), three for *CSN2* (A1, A2, L), two for *CSN1S2* (A, B), three for *CSN3* (A, B, K), four for *LALBA* (A, B, E, F), and three for *LGB* (B, B1, K). *CSN3^K^*, *LALBA^F^*, *LGB*^*B*1^ and *LGB^K^* are undescribed so far.

### 4.1. Sequence Polymorphisms and Milk Proteins in Beninese Cattle

#### 4.1.1. CSN1S1

The detection of *CSN1S1^B^* and *CSN1S1^C^* substantiates the previous identification of α_s1_-CN^B^ and α_s1_-CN^C^ at protein level in Beninese cattle [18]. The lower distribution of the protein variant *CSN1S1^C^* in the taurine breeds (in contrast to the crossbreeds) is consistent with results in other African cattle breeds [9,17,19]. The association of the *CSN1S1^C^* variant with impaired immunoreactivity in humans (in contrast to *CSN1S1^B^*) suggests its conservation through the valorization of milk from zebu and crossbreed animals for human health [67]. We are not able to elucidate the rare allele α_s1_-CN^X^ identified by Moazami-Goudarzi et al. [18] by protein electrophoresis in the Borgou cattle. Therefore, analyses on protein level using milk samples is necessary to infer the protein allele α_s1_-CN^X^ and to clarify its DNA sequence difference. The isoelectric focusing method described by Giambra et al. [68] with adjacent mRNA or DNA sequencing can be a useful approach in this regard. 

Seven SNP within the 5′-UTR of *CSN1S1* represent the causal polymorphisms of the *CSN1S1* promoter alleles 1, 2, 3 and 5, as previously described by Prinzenberg et al. [43] and Ibeagha-Awemu et al. [42]. However, only the *CSN1S1*Prom^1^ and *CSN1S1*Prom^2^ were fully identified in our samples. The *CSN1S1*Prom^3^ and *CSN1S1*Prom^5^ diverge from our sequences with the absence of some characteristic polymorphisms.

Moreover, we observed various effects of the SNP located in the 5′- and 3′-UTR of the *CSN1S1* gene on TFBS and miRNA targets. Similar impacts of SNP in the *CSN1S1* flanking regions on TFBS and milk protein content have been previously reported in cattle [42,43] and in small ruminants [69].

#### 4.1.2. CSN2

The distributions of *CSN2*^*A*1^ and *CSN2*^*A*2^ in the Somba and crossbreed populations are in line with previous findings [18,19]. Some differences in the allele frequencies of the two alleles may be due to a greater local limitation in the samples of Moazami et al. [18]. In contrast to our study, the authors only studied Lagune and Borgou animals from the regions of Porto Novo and Borgou, respectively. Due to a wider distribution of the animals sampled in our study in Benin and an associated low relationship between the animals, it is possible that we were able to present the more current allele frequency for these casein variants in Beninese cattle. However, we did not detect the variant *CSN2^I^* reported by Ceriotti et al. [19] in the Borgou. Interestingly, we found *CSN2^L^* in a crossbreed animal. The protein variant has recently been described at low frequency (0.02) in Gyr cattle by Gallinat et al. [15]. Said Ahmed et al. [9] similarly observed this protein variant in 10% of their genotyped Butana cattle. However, recent analysis of the 1000 Bull Genomes considering 14 different taurine breeds did not detect *CSN2^L^*. In consequence, our findings reinforce the specificity of *CSN2^L^* to indicine cattle. Further rare variants *CSN2^B^* and *CSN2^D^* previously reported in other African taurine (Baoulé, N’Dama) and indicine breeds (Sudanese Fulani, zebu Shuwas Arab) [17] were not identified in our study.

In contrast to the other investigated milk protein genes, we detected no SNP in the 5′-UTR and only one SNP in the 3′-UTR of *CSN2*. The sequence analyses suggested that the latter SNP (rs440770944C>A) induces the binding of the p63 TF. However, no influence of the p63 TF on milk protein gene transcription has been previously reported. In addition, the impact of SNP positioned in non-coding regions of the *CSN2* gene has been rarely reported in cattle. Only one SNP located in the 5′-upstream region (g.1311T>C) of the caprine *CSN2* was associated with a lack of β-CN in milk [70].

#### 4.1.3. CSN1S2

In contrast to the other milk protein genes, no previous study reported the distribution of the protein variants of *CSN1S2* in the Beninese cattle breeds. The gene has a low polymorphic level in cattle, and consequently, it was only marginally addressed [71]. The detection of the *CSN1S2^A^* in 99% of the investigated animals confirm its prevalence in cattle [14]. In addition, the protein variant *CSN1S2^B^* is detected in only one crossbreed animal, in agreement with its specificity to indicine cattle [71]. Ibeagha-Awemu et al. [71] observed this variant in European cattle breeds influenced by zebu introgression. In addition, *CSN1S2^D^* and *CSN1S2^E^* reported in low frequencies in some European and Iranian cattle breeds, respectively, were not found in this study [14,15,71]. 

Only one SNP (rs109900747T>C) was observed in the 5′-UTR of *CSN1S2*, whereas Said Ahmed et al. [9] reported two other SNP in this region. The alternative allele rs109900747C alters several TFBS, but promotes the c-Ets-1 TFBS and the target site of the bta-miR-452 miRNA. Similar results were reported by Kishore et al. [39] in the 5′-flanking region of *CSN1S2* in Indian zebu cattle. Furthermore, two SNP in the 3′-UTR induce the deletion and/or the substitution of several miRNA binding sites. Some of these miRNA were associated with milk production traits including milk fat metabolism [72,73]. Therefore, the impact of the alteration/substitution of miRNA target sites from SNP in 3′-UTR of *CSN1S2* would be interesting for investigations in an experimental study.

#### 4.1.4. CSN3

The identification of *CSN3^A^* and *CSN3^B^* in Beninese cattle breeds is in accordance with previous results [18,19]. In addition, the high frequencies of the *CSN3^B^* causative SNP supports the known prevalence of this variant in African taurine [14,17]. Further variants *CSN3^A^*^1^, *CSN3^H^* and *CSN3^J^* as previously reported in African indicine and taurine breeds, respectively, were not detected in our samples. However, the SNP (BTA6:85656526C>T) causing p.Ala66Val is interesting, as it has never been reported before. The *CSN3* is highly polymorphic with several variants (e.g., A^1^, E, F^2^, H, I) due to only one SNP, respectively [14]. We name this new variant *CSN3^K^* following the previous nomenclature in cattle [13,14].

We identified a SNP (BTA6:85658779C>A) in the 3′-UTR of *CSN3*, which has not been described before. This SNP abrogates the target site of the bta-miR-193a-3p. Additionally, the 3′-UTR of *CSN3* displays two other SNP including the rs109787476T>A, which affect miRNA target sites. Said Ahmed et al. [9] similarly reported several SNP affecting miRNA binding in this region in the Butana cattle, but none of these SNP was identified in our study. Moreover, a SNP in the non-coding region of the *CSN3* gene was associated with milk production traits [14,74]. In consequence, the detected polymorphisms in the 3′-UTR of *CSN3* are of interest as they may affect the expression of the gene, and consequently, they influence the technological quality of milk in Beninese cattle. Indeed, the importance of the *CSN3* gene in cheesemaking properties of milk is well established [14].

#### 4.1.5. LALBA

In *LALBA*, we observed the predominance of the variant *LALBA^B^*, while *LALBA^A^* was only found in some crossbreed animals. These findings are in agreement with reports addressing Beninese cattle as well as other African cattle breeds [9,14,17,18]. No taurine animal presents the *LALBA^A^* variant in our samples. This variant is more prevalent in indicine cattle, but has been previously reported in low frequency in African taurine [17,18]. The identification of the rare variant *LALBA*^E^ in the Beninese cattle supports its recent description in the African zebu Butana as well as in some European and Asian breeds [9,52]. Tetens et al. [52] associated the presence of this variant in the Hinterwälder taurine breed with prior indicine introgression. The presence of this variant in some Somba cattle originated from zebu, considering the existing indicine introgression in the Somba cattle breed [45]. Nevertheless, the evaluation of the *LALBA^E^* distribution in other breeds is recommended. Additionally, we identified the missense SNP rs714688595C>T in Somba and crossbreed cattle. This polymorphism is known and annotated in the Ensembl variant table for the bovine *LALBA* gene. However, we found no study describing the p.Arg10Trp substitution in α-LA caused by this SNP. Indeed, α-LA^A^ diverges from α-LA^B^ by the AA replacement p.Arg10Gln. Further known AA exchanges in α-LA include the variants C (p.Gln>Glu, unknown position), D (p.Gln65His) and E (p.Ile41Val) [14,75,76], whereas we could not identify the causing SNP on DNA-level in our animal material. Therefore, we believe that SNP rs714688595C>T leading to p.Arg10Trp characterizes a new protein variant *LALBA^F^*. 

The detection of the SNP rs718675014C>T and rs110359174A>G in the 3′-UTR corroborates previous findings in the Butana cattle [9]. In addition, the functional effects of these polymorphisms in the bta-miR-143 and bta-miR-2467-3p, respectively, concur with the findings of Said Ahmed et al. [9]. However, we retrieved less miRNA binding sites or targets/seeds affected by the SNP. Several SNP were observed in the 5′-UTR of the *LALBA* gene, but they were not addressed in previous studies (Table S3). Many of these SNP are noteworthy as they affect TFBS or miRNA-binding sites, with potential influences on the differential expression of *LALBA* in the Beninese cattle.

#### 4.1.6. LGB

The high frequency of *LGB^B^* (corresponding to the reference sequence, see Table 1) supports previous findings in Beninese as well as in other African cattle breeds [17,18]. In contrast to Moazami-Goudarzi et al. [18] and Ceriotti et al. [19], we cannot confirm the presence of *LGB^A^*. This variant is due to two missense SNP rs110066229G>A (p.Gly64Asp) and rs109625649C>T (p.Ala118Val), and the two synonymous SNP rs110180463C>T (p.Asn63) and rs110641366T>C (p.Asn88) [40,77]. As we could only identify the SNP rs109625649C>T and rs110641366T>C in our animal material, we propose that this is an intermediate *LGB* form, not described until now. Moazami-Goudarzi et al. [18] used isoelectric focusing to segregate β-LG^A^ from β-LG^B^, whereas it is not mentioned if the differences in the electrophoretic separation is caused by changes in the isoelectric point due to both amino acid exchanges p.Gly64Asp and p.Ala118Val, or only due to one of the amino acid exchanges. In the study by Ceriotti et al. [19], the typing of the protein variant *LGB^A^* was based on the visualization of the SNP rs109625649C>T using the PCR-restriction fragment length polymorphism (RFLP) method. The second SNP rs110066229G>A was not analyzed using this method [78]. Therefore, it is possible that the animal material examined by Ceriotti et al. [19] did not contain the actual variant *LGB^A^* but, as in our case, the allele *LGB*^*B*1^. Ganai et al. [40] observed a complete linkage disequilibrium (LD) between the causal SNP of *LGB^A^* and *LGB^B^* in Dutch Holstein Friesian cows. Therefore, the omission of the SNP rs110066229G>A in the Beninese cattle breeds is unprecedented and may suggest the breakage of LD between the two major causal SNP of *LGB^A^* and *LGB^B^* in the Beninese cattle. Considering the existing information, we annotate the intermediate form of the *LGB* gene sequence caused by the missense SNP rs109625649C>T (p.Ala118Val) and the synonymous SNP rs110641366T>C (p.Asn88) as *LGB*^*B*1^. 

Another new protein variant detected in this study is the β-LG^K^. This variant is due to an insertion at BTA11:103257980 in exon 3 altering the reading frame, and inducing a premature stop codon (p.Thr92Asnfs*13). The mutated protein is shortened by 74 AA (46%) and probably lacks domains that are essential for normal protein function. Although peptide deletions or insertions characterize some milk protein variants in ruminants, the loss of a large peptide sequence as observed in this study is uncommon [14,79,80]. Firstly, to our knowledge, no peptide skipping has been previously reported in the ruminant β-LG protein. Secondly, the largest peptide deletion identified in cattle associated the α_s1_-CN^A^ with the loss of 13 AA caused by a single T>A SNP [81]. Further deletions of peptide sequence in cattle were associated with the α_s1_-CN^H^ (peptide 51 to 58) in Kuri (an African crossbreed) and α_s2_-CN^D^ (peptide 51 to 59) in European breeds due to exon skipping events [17,79]. In goats, the deletions of larger peptide sequences are more frequent with significant effects on milk protein genes. For instance, the caprine α_s1_-CN*^F^* (37 AA deleted) as well as several null alleles (α_s1_-CN^O1^, α_s1_-CN^O2^, α_s2_-CN^O^, β-CN^O^) were associated with low or null expression of casein genes, respectively [79,82]. Therefore, we presume a high impact of the observed peptide skipping in the β-LG protein due to the described insertion in *LGB* exon 3. With regard to the indirect effect of the *LGB* gene on the total casein content [83,84], the variant *LGB^K^* could affect animal milk composition and yield. The low frequency of the variant in the investigated population suggest a relative recent occurrence of the variant or indirect negative selection against animals carrying this variant. Further investigations should evaluate the functional impact of the *LGB*^K^ variant. Moreover, this protein variant is interesting given the high allergenic potential of β-LG, a milk protein missing in human milk [14]. For instance, camel milk containing no β-LG is recommended as an alternative milk protein source for cow milk protein allergy patients [85,86,87]. Similar observations were also made for the bovine *CSN1S1*^G^ and several null casein alleles in goats [83,88]. 

In addition to the missense and frameshift variants, we detected two previously not described synonymous SNP. Further investigations are imperative to clarify the impact of the new synonymous variants in the *LGB* gene. Indeed, Cartegni et al. [89] revealed potential effects of synonymous SNP on splicing. Various synonymous SNP are also linked to specific protein variants (e.g., *CSN2*^*A*2^, *CSN2*^*A*3^, *CSN3*^*A*’^) [14]. 

The unique polymorphism observed in the 3′-UTR of *LGB* induces the loss of a bta-miR-7860 target site (Table 5). Regarding the polymorphisms in 5′-UTR of the *LGB* gene, two SNP promote the gain of TFBS, whereas the third alters a TFBS and the target sites of several miRNA. The SNP rs41255683G>C is in close proximity (at 34 bp) to SNP g.-215C>A (BTA5:103255964) causing a low expression level of *LGB* in cattle [90]. We were not able to detect the g.-215C>A SNP, but it seems to be rare, as it was similarly not found by Ganai et al. [40], and is also not referenced in the Ensembl variant table. Nevertheless, we mapped the SNP rs41255679C>G in the 5′-upstream region of *LGB*. This polymorphism was reported within the TF activator protein-2 (AP-2), and associated with differential expression of the *LGB*^*A*^ and *LGB*^*B*^ protein variants [40,41]. These findings evidence the necessity to demonstrate the potential effects of SNP positioned in 5′-upstream regions on milk protein genes.

### 4.2. Casein Haplotypes

The presence of the haplotype B-A1-A-B (corresponding to *CSN1S1-CSN2-CSN1S2-CSN3*) in about 50% of the Lagune and Somba cattle (Table 4), is in agreement with previous findings in the Beninese and African taurine breeds [17,18,42]. Bonfatti et al. [21] related haplotypes including *CSN2*^*A*1^ and *CSN3^B^* with increased α_s1_- and κ-CN milk contents, and reduced β- and α_s2_-CN concentrations. *CSN2*^*A*1^ is negatively correlated with milk digestibility and human health [11,91]. In addition, haplotypes with the *CSN2*^*A*1^ were associated with low milk and protein contents [20,22]. Therefore, the low milk performance of the Beninese taurine cattle is possibly due to the high frequency of the haplotype B-A1-A-B [7]. Inversely, a high frequency of the haplotype B-A2-A-A was described for Holstein and several European taurine breeds [20,60], which favorably supports the importance of the *CSN2*^*A*2^ in bovine milk production. The latter haplotype was identified in low frequency in African taurine and the Sudanese Butana cattle [9,17,42], but not in the present study. Nevertheless, the distribution of the haplotype B-A2-A-B in moderate frequencies over all breeds, is interesting as it may be associated with appreciable milk performances and should be included in future Beninese cattle improvement strategies. This haplotype is similarly predominant in the Brown Swiss cattle population [20,60]. Moreover, we estimated a high frequency of the haplotypes B-A1-A-A and C-A2-A-B in the crossbreeds. Remarkably, the distribution of the latter haplotype in crossbreeds is linked to the high occurrence of *CSN1S1^C^* in indicine cattle. However, this haplotype differs with *CSN3^B^* from the most frequent haplotypes in indicine cattle (C-A2-A-A) [9,17]. These results suggest the presence of both indicine and taurine imprints in caseins regions in the crossbreeds. With regard to their association with milk performances, Boettcher et al. [20] related haplotypes comparable to B-A1-A-A and C-A2-A-B (i.e., excluding the *CSN1S2* gene) with higher milk protein and fat contents, and with lower milk yield in Italian Holstein and Brown Swiss Cattle. Considering that the *CSN1S2^A^* protein variant is the most prevalent in European taurine breeds, we assume that the haplotypes described by the latter authors correspond to B-A1-A-A and C-A2-A-B, and in consequence, we expect the same effects [13]. Finally, the casein haplotype B-A1-A-A was rare in African cattle, but dominant in British Friesian and German Black Pied Cattle [17,42,44,60]. 

## 5. Conclusions

The current study confirmed milk protein variants in Beninese cattle breeds as previously identified in commercial or outbred populations. As a novelty based on the analyses of sequence data, unknown polymorphisms including SNP and InDel, were detected. In this regard, the identified new milk protein variants are located in the *CSN3*, *LALB*A and *LGB* genes. Hence, the Beninese cattle carry favorable alleles as well as haplotypes, which can be used in genetic improvement programs for milk yield and milk quality of various breeds.

## Figures and Tables

**Table 1:** Reference gene sequences used to investigate milk protein gene polymorphisms in the Beninese indigenous cattle breeds.

Gene Name	Gene Sequence			
	Sequence ID	Location	Strand ^1^	Exons (*n*)
***CSN1S1***	ENSBTAG00000007695	6:85410518:85429868	+	19
***CSN2***	ENSBTAG00000002632	6:85448564:85458344	−	9
***CSN1S2***	ENSBTAG00000005005	6:85529305:85549156	+	18
***CSN3***	ENSBTAG00000039787	6:85645254:85659526	+	5
***LALBA***	ENSBTAG00000005859	5:31182832:31213745	+	4
***LGB***	ENSBTAG00000014678	11:103255224:103264876	+	7

^1^ + = forward strand, − = reverse strand.

**Table 2:** Number and percentage distribution (in brackets) of the sequence variants (SNP, InDel) detected within the exon sequences, flanking intron sequences and parts of the 5′-flanking region of the six milk protein genes (*CSN1S1*, *CSN2*, *CSN1S2*, *CSN3*, *LALBA*, *LGB*) in Beninese indigenous cattle breeds.

	*CSN1S1*	*CSN2*	*CSN1S2*	*CSN3*	*LALBA*	*LGB*
	*n* (%)					
**SNP**	20 (8.06)	8 (5.83)	26 (7.53)	15 (5.23)	3 (11.54)	1 (2.22)
**InDel ^1^**	228 (91.94)	114 (94.17)	313 (92.47)	275 (94.77)	23 (88.46)	44 (97.78)
**Total**	248	120	332	287	26	45

^1^ InDel = insertions/deletions.

**Table 3:** Description (ID, location and allele frequencies) of the variants causing protein polymorphisms of the six milk protein genes (*CSN1S1*, *CSN2*, *CSN1S2*, *CSN3*, *LALBA*, *LGB*), their effects on the protein sequence and the designations of consequent protein variants, and frequencies within the different Beninese local breeds and across all investigated cattle.

Genes	SNP			Gene Transcript				Variant Frequency			
	ID ^1^	BTA:bp ^2^	Allele	Exon	Protein ^3^	Amino Acid ^4^	Variant ^5^	Crossbreed (*n* = 27)	Lagune (*n* = 20)	Somba (*n* = 20)	Total (*n* = 20)
***CSN1S1***	rs43703010	6:85427427	A	17	192 (207)	Glu	B	0.62	0.97	0.93	0.81
			G			Gly	C	0.38	0.03	0.08	0.19
***CSN2***	rs715383373	6:85450908	T	7	197 (247)	Val	A1	0.97	1.00	1.00	0.99
			C			Ala	L	0.03	0.00	0.00	0.01
	rs43703011	6:85451298	A	7	67 (117)	His	A1	0.39	0.80	0.63	0.57
			C			Pro	A2	0.61	0.20	0.37	0.43
***CSN1S2***	rs441966828	6:85533780	C	3	8 (23)	Ser	A	0.98	1.00	1.00	0.99
			T			Phe	B	0.02	0.00	0.00	0.01
***CSN3***	ss7626433428	6:85656526	C	4	66 (87)	Ala	B	1.00	0.75	1.00	0.97
			T			Val	K	0.00	0.25	0.00	0.03
	rs43703015	6:85656736	T	4	136 (157)	Ile	B	0.50	0.75	0.80	0.72
			C			Thr	A	0.50	0.25	0.20	0.28
	rs43703016	6:85656772	C	4	148 (169)	Ala	B	0.50	0.50	0.80	0.69
			A			Asp	A	0.50	0.50	0.20	0.31
***LALBA***	rs714688595	5:31184282	C	1	10 (58)	Arg	B	0.96	1.00	0.88	0.94
			T			Trp	F	0.04	0.00	0.13	0.06
	rs722550244	5:31184283	G	1	10 (58)	Arg	B	0.88	1.00	1.00	0.95
			A			Gln	A	0.12	0.00	0.00	0.05
	rs465119286	5:31184696	A	2	41(89)	Ile	B	0.88	1.00	0.95	0.93
			G			Val	E	0.12	0.00	0.05	0.07
***LGB***	rs109625649	11:103259232	C	4	118 (134)	Ala	B	0.89	0.50	0.81	0.80
			T			Val	B1	0.11	0.50	0.19	0.20
	ss7626433430	11:103257980	-	3	Thr92	-	B	0.96	1.00	1.00	0.98
			A		Thr92Asnfs*13	-	K	0.04	0.00	0.00	0.02

^1^ Variant ID: the rs# accession number of known variants were retrieved from the Ensembl database; the ss# accession number of the new variants identified from our sequencing were assigned by the European Variation Archive; ^2^ bp = SNP position according to the ARS-UCD1.2 genome map; ^3^ Amino acid (AA) position in mature protein. The corresponding AA positions in the protein sequence including signal peptide are given in parentheses; ^4^ Ala: Alanine, Arg: arginine, Asp: Aspartic acid, Gln: glutamine, Glu: glutamic acid, Gly: Glycine, His: histidine, Ile: isoleucine, Phe: Phenylalanine, Pro: proline, Ser: serine, Thr: Threonine, Trp: Tryptophan, Val: Valine; ^5^ Milk protein variants were inferred following previous nomenclature [9,14,15,52]. The underlined variants have been newly annotated.

**Table 4:** Frequencies of the bovine casein haplotypes (*CSN1S1-CSN2-CSN1S2-CSN3*) in the different Beninese breeds and across all investigated cattle.

Haplotypes ^1^	Crossbreed (*n* = 27)	Lagune (*n* = 20)	Somba (*n* = 20)	Total (*n* = 20)
**B-A1-A-B**	0.01	0.50	0.47	0.39
**B-A2-A-B**	0.20	0.18	0.25	0.22
**B-A1-A-A**	0.39	0.29	0.20	0.20
**C-A2-A-A**	<0.01	<0.01	0.02	0.19
**C-A2-A-B**	0.38	<0.01	0.02	<0.01

^1^ Only haplotypes with a minimum frequency of 5% in one breed are considered.

**Table 5:** Potential effects of SNP positioned within the 5′- and 3′-untranslated regions (UTR) of bovine milk protein genes on the binding of micro-RNA (miRNA) and transcription factors (TF) in Beninese cattle breeds.

Gene	Location (BTA:bp ^1^)	Allele (ref/alt) ^2^	ID ^3^	Position	miRNA		TF	
					Loss ^4^	Gain ^4^	Loss ^4^	Gain ^4^
*CSN1S1*	6:85411136	C/T	rs379920406	5′-UTR	-	bta-miR-2291	-	-
	6:85411147	G/A	rs517257790	5′-UTR	-	-	-	-
	6:85411192	C/T	rs520777414	5′-UTR	-	-	-	-
	6:85411221	A/G	rs133040184	5′-UTR	bta-miR-133c	bta-miR-9-5p	-	-
	6:85411236	A/G	rs137119956	5′-UTR	bta-miR-2446	-	E2F-6	-
	6:85411427	A/C	rs135615686	5′-UTR	-	-	RAR	-
	6:85411578	A/G	rs109817504	5′-UTR	-	-	ATF-3; B-ATF; c-Jun; SMARCC1; SMARCC2	-
	6:85411618	C/G	rs109757609	5′-UTR	bta-miR-2284e	bta-miR-7864	-	Sp1; IRF-4
	6:85411677	G/A	rs134534951	5′ UTR	-	-	STAT5A	-
	6:85411780	T/C	rs110163054	5′-UTR	bta-miR-15a; bta-miR-562	-	-	-
	6:85413195	C/T	rs110899610	5′-UTR	bta-miR-2325b; bta-miR-2420; bta-miR-453	bta-miR-376a	-	-
	6:85428962	G/A	rs466704456	3′-UTR	bta-miR-874	bta-miR-219-5p; bta-miR-2355-3p	-	-
	6:85429024	C/T	ss7626432754	3′-UTR	-	-	-	-
	6:85429077	A/G	rs435231328	3′-UTR	bta-miR-338	-	-	-
	6:85429241	A/G	rs211141048	3′-UTR	bta-miR-380-3p	-	SMAD4	-
	6:85429252	C/A	rs716970086	3′-UTR	bta-miR-371	-	SMAD4	-
*CSN2*	6:85449252	C/A	rs440770944	3′-UTR	bta-miR-2464-3p	-	-	p63
*CSN1S2*	6:85531897	T/C	rs109900747	5′-UTR	-	bta-miR-452	Sp1; HNF-4alpha; HNF-4gamma; FOXO1	c-Ets-1
	6:85548443	A/G	rs211156498	3′-UTR	bta-miR-2318; bta-miR-2479; bta-miR-2480	bta-miR-2463; bta-miR-452	CP2-L1	-
	6:85548520	A/G	rs109274107	3′-UTR	-	bta-miR-1388-3p	-	-
*CSN3*	6:85656841	T/A	rs109787476	3′-UTR	bta-miR-496	bta-miR-2284w	ZNF274	
	6:85658779	C/A	ss7626433382	3′-UTR	bta-miR-193a-3p	-	-	-
	6:85658873	G/A	rs134516686	3′-UTR	-	-	-	-
*LALBA*	5:31183709	G/C	rs444727593	5′-UTR	-	bta-miR-2475	-	-
	5:31183736	G/A	rs458466372	5′-UTR	bta-miR-2284r; bta-miR-2284s	-	-	-
	5:31183766	T/C	rs471361585	5′-UTR	-	bta-miR-2370-5p	-	-
	5:31183789	A/G	rs440012037	5′-UTR	bta-miR-7864		-	-
	5:31183806	C/T	rs717249686	5′-UTR	-	-	-	KLF4
	5:31183848	C/A	rs460157851	5′-UTR	-	bta-miR-2472; bta-miR-6517	-	AP-2alpha; IRF-9, STAT2;STAT1
	5:31183921	A/G	rs448925171	5′-UTR	bta-miR-2325c; bta-miR-6120-3p	bta-miR-218; bta-miR-2452	-	-
	5:31183924	T/C	rs719631407	5′-UTR	bta-miR-6120-3p	bta-miR-218	-	-
	5:31184082	T/C	rs462561324	5′-UTR	-	-	-	-
	5:31185981	C/T	rs718675014	3′-UTR	-	bta-miR-2425-5p; bta-miR-2467-3p	N-Myc; N-Myc	c-Myc
	5:31186027	A/G	rs110359174	3′-UTR	bta-miR-143	-	-	-
*LGB*	11:103255847	G/A	rs516356159	5′-UTR	-	-	-	RCoR2
	11:103255918	C/T	rs41255682	5′-UTR	bta-miR-1777a; bta-miR-2454-5p	-	SIX5	-
	11:103255930	G/C	rs41255683	5′-UTR	-	-	-	STAT1; STAT3
	11:103260824	G/A	rs1116405113	3′-UTR	bta-miR-7860	-	-	-

^1^ bp = SNP position; ^2^ ref/alt = reference/alternate allele, ^3^ Variant ID: the rs# accession number of known variants were retrieved from the Ensembl database; the ss# accession number of the new variants identified from our sequencing were assigned by the European Variation Archive; ^4^ loss of binding, gain of binding.

## Data Availability

All the DNA polymorphisms identified from our sequencing are submitted to the European Variation Archive (EVA) and are openly accessible at https://wwwdev.ebi.ac.uk/eva/?eva-study=PRJEB47999, accessed on 13 October 2021, [51]. Further data supporting the results of this article are presented in the Supplementary files.

## Supplementary Materials

The following are available online at https://www.mdpi.com/article/10.3390/genes12111702/s1, Table S1: Primer sequences utilized for the sequencing of the six milk protein genes (*CSN1S1*, *CSN2*, *CSN1S2*, *CSN3*, *LALBA*, *LGB*) in the Beninese indigenous cattle breeds; Table S2: List of the bovine miRNA and their corresponding seed sequences retrieved from the TargetScan website and used in “targetscan_60.pl” tool for the detection of miRNA target sites; Table S3: List of the polymorphisms detected within the exon sequences, flanking intron sequences and parts of the 5′-upstream regions of milk protein genes (*CSN1S1*, *CSN2*, *CSN1S2*, *CSN3*, *LALBA*, *LGB*) in Beninese indigenous cattle breeds; Table S4: Distributions of variant types (according to their positions and consequence) detected within each of the six milk protein genes (*CSN1S1*, *CSN2*, *CSN1S2*, *CSN3*, *LALBA*, *LGB*) and total in Beninese indigenous cattle breeds; Table S5: Functional consequence of the frameshift insertion of nucleotide A at BTA11:103257980 on the translational reading frame and on mature protein.
